# A Bifunctional BiOBr/ZIF-8/ZnO Photocatalyst with Rich Oxygen Vacancy for Enhanced Wastewater Treatment and H_2_O_2_ Generation

**DOI:** 10.3390/molecules28062422

**Published:** 2023-03-07

**Authors:** Xiao Han, Tianduo Zhang, Yang Cui, Zhaoyang Wang, Ruoyu Dong, Yuhan Wu, Cuiwei Du, Ruyan Chen, Chongfei Yu, Jinglan Feng, Jianhui Sun, Shuying Dong

**Affiliations:** Key Laboratory of Yellow River and Huai River Water Environment and Pollution Control, Ministry of Education, Henan Key Laboratory for Environmental Pollution Control, School of Environment, Henan Normal University, Xinxiang 453007, China

**Keywords:** BiOBr/ZIF-8/ZnO, H_2_O_2_ production, S-scheme heterojunction, oxygen vacancy

## Abstract

Photocatalytic technology is considered an ideal approach for clean energy conversion and environmental pollution applications. In this work, a bifunctional BiOBr/ZIF-8/ZnO photocatalyst was proposed for removing phenols in wastewater and generating hydrogen peroxide. Insights from scanning electron microscopy measurements revealed the well-dispersion of ZIF-8/ZnO was on the BiOBr layer, which could effectively prevent agglomeration of ZIF-8 and facilitate the separation of carriers. In addition, the optimal H_2_O_2_ yield of the BiOBr/ZIF-8/ZnO sample could reach 116 mmol·L^−1^·g^−1^ within 2 h, much higher than that of pure BiOBr (with the value of 82 mmol·L^−1^·g^−1^). The optimal BiOBr/ZIF-8/ZnO sample could also remove 90% of the phenol or bisphenol A in 2 h, and its kinetic constants were 3.8 times and 2.3 times that of pure BiOBr, respectively. Based on the analysis of the various experimental characterizations, the photocatalytic mechanism of the S-scheme BiOBr/ZIF-8/ZnO composite for the degradation of phenolic pollutants and generation of H_2_O_2_ was proposed. The formation of the heterojunction and the oxygen vacancy work together to significantly improve its photocatalytic efficiency. In addition, the BiOBr/ZIF-8/ZnO catalyst has a certain impact on the degradation of phenol in actual wastewater, providing a way to effectively remove refractory pollutants and generate H_2_O_2_ in actual water.

## 1. Introduction

Environmental contamination and energy problems are two major issues that need to be solved urgently [1]. In recent years, hydrogen peroxide (H_2_O_2_), as a strong oxidant, has been regarded as an ideal fuel cell carrier for hydrogen substitutes because of the lack of secondary pollution [2,3]. Generally speaking, H_2_O_2_ is produced by the oxidation of the anthraquinone method and the electrocatalytic method. However, the traditional method has a high cost and complex pathway [4,5,6]. Hence, the development of an efficient and energy-saving method is urgent in the current research.

Photocatalytic technology is a clean and low-cost method for treating refractory pollutants and producing H_2_O_2_. In recent years, most of the modified carbon-based materials are used in the photocatalytic production of H_2_O_2_ [7,8,9]. For example, Wang et al. [8] prepared a nitrogen-vacancy CNKx by KOH modified, which could photocatalytic produce 101 μmol of H_2_O_2_ within 60 min. In another interesting work, Jourshabani et al. [7] prepared an oxygen-doped carbon nitride nanosheet, which removed both tetracycline and rhodamine B from water within 15 min. The authors also proved that a certain amount of H_2_O_2_ can be stably generated without sacrificing agents. Zhang et al. [10] constructed porous 1, 3, 5-trihydroxybenzene that substituted g-C_3_N_4_ (PTBCN) photocatalysts which has an excellent removal effect of rhodamine B (RhB) 6.56 times that of pure g-C_3_N_4_, and the photocatalytic rate constant of H_2_O_2_ is 3.17 times that of pure g-C_3_N_4_. However, in addition to carbon-based materials, metal oxides or other semiconductor materials have also been used for the photocatalytic production of H_2_O_2_. Zhang et al. [11] prepared ZnO/COF (TaPa-Cl) catalysts by employing the electrostatic self-assembly method, and the maximum yield of H_2_O_2_ was 2443 μmol·g^−1^·h^−1^, 3.3 and 8.7 times that of pure ZnO and COF. Du et al. [12] synthesized Zn_2_In_2_S_5_ grown on N-doped hollow carbon spheres by using in situ hydrothermal methods, which could produce a certain amount of H_2_O_2_ when the aromatic pollutants in water were removed.

Bismuth-based materials are often used in photocatalytic experiments because of their open structure and suitable band gap. BiOBr was considered an ideal catalyst support due to its low cost, high stability, visible light response, and diverse microstructure. However, the high carrier recombination rate limits its practical application, so modification and recombination with other catalysts are regarded as effective strategies [13]. Deng et al. [14] grew CuO quantum dots on the {001}-facet exposed BiOBr nanoflakes to construct a p-n heterojunction. The prepared heterojunction could easily remove Congo red and bisphenol A (BPA) in the water. Moreover, Liu et al. [15] prepared BiVO_4_/BiOBr Z-type heterojunctions with oxygen vacancies, which could effectively degrade oxytetracycline, and inferred three degradation pathways. Chuaicham et al. [16] fabricated carbon quantum dots/saponite/BiOBr by using the hydrothermal method, demonstrating excellent ciprofloxacin degradation performance under visible light. Wu et al. [17] doped Mo into BiOBr nanosheets to narrow the band gap of BiOBr, thus obtaining a better visible light response. From the acquired results, it was shown that the modified BiOBr could remove sulfanilamide efficiently, while its kinetic constant was 2.3 times that of pure BiOBr.

Metal-organic frameworks (MOFs) are porous coordination polymers with organic ligands and metal centers, which have applications in gas storage, water treatment, catalysis, adsorption, and separation [18]. Zeolite imidazolate frame-8 (ZIF-8) is a typical MOF material composed of metal atoms Zn^2+^ and 2-methylimidazole. ZIF-8 has a unique structure, which is attractive for applications in adsorption and photocatalytic materials, making it a high-quality material for wastewater treatment [19]. However, ZIF-8 particles can easily agglomerate, which limits their application in water treatment. At present, scientists have prepared a new type of catalyst by using ZIF-8 and other composite materials to solve this problem. Particularly, Yang et al. [20] used ZIF-8 as the precursor to prepare ZnO/ZIF-8 hybrid photocatalysts by adjusting the concentration of AgNO_3_ in the intermediate solution. The prepared ZnO/ZIF-8 composite could remove more than 98% within 12 min of UV light irradiation. Additionally, Zhou et al. [21] synthesized an Ag/AgCl@ZIF-8 modified g-C_3_N_4_ material configuration with various electron transfer pathways, which could remove 87.3% of levofloxacin in 60 min assisted by peroxymonosulfate. Qiu et al. [22] prepared Cd_0.5_Zn_0.5_S@ZIF-8 materials by using a mild hydrothermal method, which revealed a strong photocatalytic reduction ability for Cr(VI). In addition, the combination of ZIF-8 and its derivatives with Bi-based materials has also been studied in wastewater treatment [23,24,25]. More specifically, Zhang et al. [26] dispersed ZIF-8 in a flower-like Bi_2_WO_6_ sheet and evaluated its photocatalytic activity by eliminating methylene blue under visible light irradiation.

Under this direction, ZIF-8 prepared as a precursor produces BiOBr/ZIF-8/ZnO compounds by employing the hydrothermal method. ZIF-8 and ZnO particles were dispersed in BiOBr lamellae, which could effectively prevent ZIF-8 from agglomerating into larger pieces and provided a pathway for electron separation at the interface. The prepared BiOBr/ZIF-8/ZnO composite could also effectively remove phenol pollutants from wastewater and generate H_2_O_2_ under the irradiation of an LED lamp. Then, the ability of phenol degradation was further studied in Yellow River water, seawater, deionized water (DI water), and tap water. Moreover, the electron transfer path and reaction mechanism in BiOBr/ZIF-8/ZnO were thoroughly investigated. To the best of our knowledge, the fabrication of BiOBr/ZIF-8/ZnO composites with the dual functions of H_2_O_2_ production and wastewater treatment has not been reported in the literature. Our work provides a solid theoretical basis for the photocatalytic treatment of wastewater and the production of H_2_O_2_ in the future.

## 2. Results and Discussion

### 2.1. Characterization

**Figure 1:**
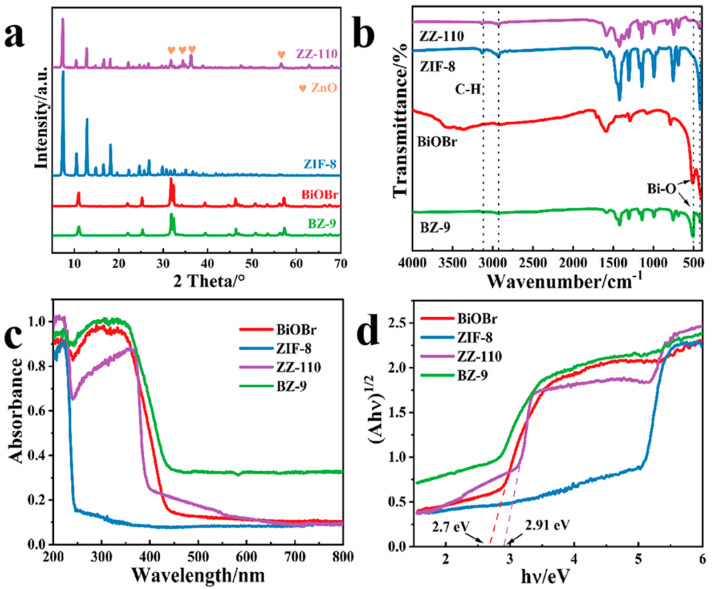
(**a**) XRD patterns of BiOBr, ZIF-8, ZZ-110, and BZ-9; (**b**) FTIR spectra of the prepared samples; (**c**) UV-vis diffuse reflectance spectra; (**d**) The band gaps of BiOBr, ZIF-8, ZZ-110, and BZ-9.

The crystal structure of ZIF-8, BiOBr, ZZ-110, and BZ-9 were probed by performing XRD measurements, as can be ascertained from Figure 1a. The peak shape and position of the prepared ZIF-8 were consistent with the simulated ZIF-8 in the literature, while its characteristic peaks were at 2θ = 7.28°, 10.24°, 12.74°, and 16.48°, which correspond to the plane of (110), (200), (211), and (220) [27]. After the hydrothermal reaction of ZIF-8 took place, the characteristic peaks of ZnO appeared in the diffraction peaks, which were located at 31.766°, 34.418°, 36.251°, and 47.535°, respectively, which could be ascribed to JCPDS No. 99-0111, indicating that ZnO was formed after hydrothermal treatment [28]. It is also worth noting that both the as-prepared BiOBr and the composites had similar characteristic peaks, which coincided with BiOBr (JCPDS No. 85-0862) [29]. The underlying reason for this phenomenon might be that the peaks of ZZ-110 were covered due to the small amount of ZZ-110 doping.

Figure 1b depicts the FT-IR spectra at 400–4000 cm^−1^ of the prepared BiOBr, ZIF-8, and BZ-9. For BiOBr and BZ-9, the peak at 501 cm^−1^ corresponded to Bi-O [30]. Furthermore, the strong absorption bands at 421 cm^−1^ coincided with the tensile vibration of Zn-N [31], while this peak was also present in BZ-9, indicating that Zn^2+^ is connected to N. The characteristic peaks recorded in the range of 600-1500 cm^−1^ in ZIF-8 could correspond to the vibrational signals of the 2-MI aromatic ring [32]. In addition, the strong absorption peaks at 994 and 1146 cm^−1^ could be consistent with the tensile vibration of the C-N band. The bands at 1458 and 1308 cm^−1^ could be interpreted by considering the methyl bending vibrations. The corresponding peaks at 3135 and 2926 cm^−1^ were also C-H in methyl and imidazole [33].

Figure 1c displays the DRS spectrum of the prepared samples, revealing the light response ability of the BiOBr, ZIF-8, ZZ-110, and BZ-9. The light absorption of the prepared catalysts was mainly recorded in the ultraviolet region. Compared with ZIF-8, other catalysts exhibited a certain degree of redshift at the absorption edge. Among them, the absorption band edge of BZ-9 was close to 500 nm, indicating that BZ-9 had a wider light response range and could respond to visible light. Thus the band gaps of ZZ-110 and BiOBr can be estimated at 2.91 and 2.70 eV, respectively, by drawing a Tauc diagram (Figure 1d), using a similar formula as our previously reported work [10].

**Figure 2:**
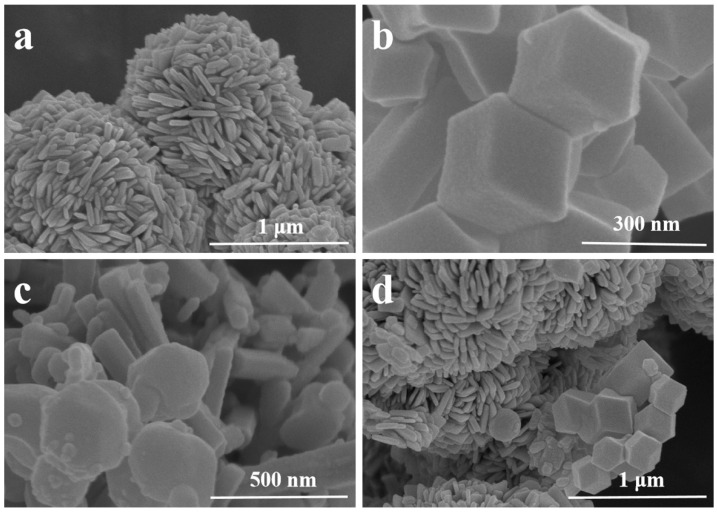
SEM image of (**a**) BiOBr; (**b**) ZIF-8; (**c**) ZZ-110; and (**d**) BZ-9.

SEM imaging was applied to present the micromorphology of the prepared samples. The acquired SEM images of the prepared BiOBr, ZIF-8, ZZ-110, and BZ-9 are illustrated in Figure 2. Pure BiOBr presented flakes stacked into circular balls with a diameter of about 1 μm. Moreover, ZIF-8 displayed a rhombic dodecahedron with a size of 300 nm, which was the same as previously reported [34]. Interestingly, after the solvothermal reaction, the edges of some ZIF-8 gradually softened and appeared spheroid, and then gradually derived a rod-like structure. The prepared BZ-9 was like BiOBr in both shape and size, while a small amount of ZZ-110 was distributed on the BiOBr layer.

**Figure 3:**
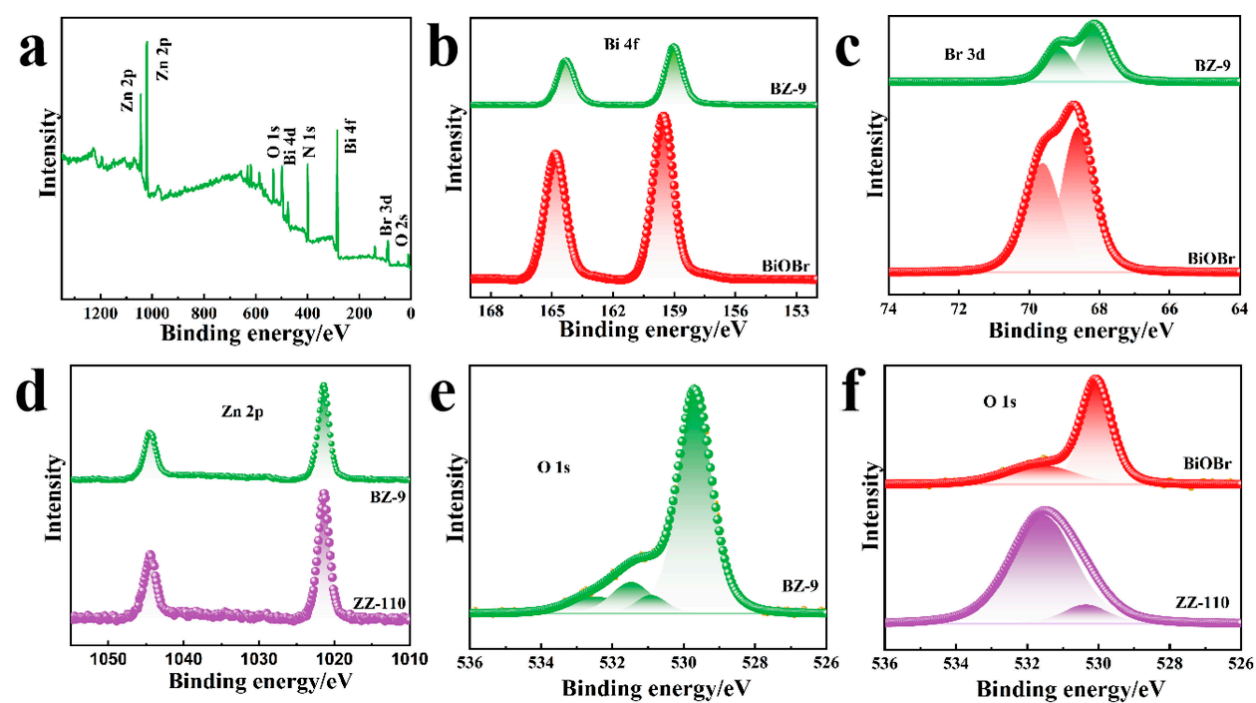
Full spectrum of BZ-9 (**a**); Characteristic spectra of Bi 4f (**b**); Br 3d (**c**); Zn 2p (**d**); and O 1s (**e**,**f**).

Figure 3 depicts a high-resolution XPS of the as-prepared materials. Figure 3a and Figure S1 present the full elemental spectra of the prepared BZ-9, ZIF-8, ZZ-110, and BiOBr, illustrating their elemental distributions. As can be clearly seen, the elements Bi, Br, O, and Zn existed in BZ-9. Figure 3b presents the high-resolution spectrum of Bi 4f in BiOBr and BZ-9. The characteristic peaks at 164.8 eV and 159.5 eV can be assigned to Bi 4f [30], while the peak of BZ-9 exhibited the blue shift of 0.5 eV. Similarly, the characteristic peak of Br in BZ-9 was also blue-shifted by 0.5 eV, compared with pure BiOBr, indicating that there was valence electron transfer between BiOBr and ZZ-110. Figure 3d and Figure S1b show the high-resolution spectrum of Zn 2p of the prepared samples. The spectrum of BZ-9 also had a slight redshift compared with ZZ-110 and ZIF-8, indicating that there was an interaction between the heterojunction. It is also worth noting that the O1s peak in BZ-9 can be divided into four distinct peaks (Figure 3e), among which the peaks at 529.7, 530.9, 531.5, and 532.4 eV were assigned to Bi-O, oxygen vacancy, C=O, and C-O, respectively [35,36,37]. However, no peaks of oxygen vacancy were observed in either BiOBr, ZIF-8, or ZZ-110, indicating that only BZ-9 possessed oxygen vacancies, which might be due to the introduction of oxygen vacancy in its reaction.

**Figure 4:**
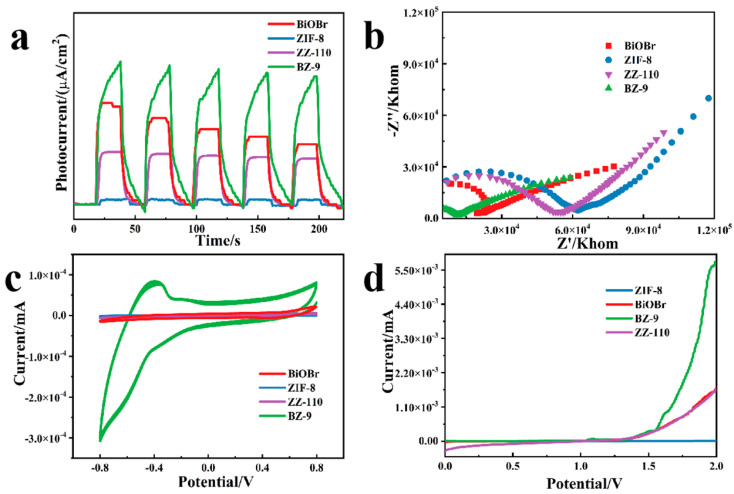
Photocurrent response (**a**); Nyquist plots of the electrochemical impedance spectra (**b**); Cyclic voltammetry curve (**c**); Linear sweep voltammetry curve of BiOBr, ZIF-8, ZZ-110, and BZ-9 photocatalysts (**d**).

Figure 4 depicts the electrochemical test results of the prepared materials, which could reflect the quantum efficiency of the catalysts. Transient photocurrent response measurements were performed by recording the current by switching the lamp on and off every 20 s, as shown in Figure 4a. As could be seen, BZ-9 had a stronger photocurrent than other catalysts, indicating that BZ-9 had stronger light absorption and carrier separation efficiency. EIS test was also used to analyze the charge transfer impedance of the catalysts. A smaller Nyquist diameter led to a faster interfacial charge transfer, and the effective separation of electron holes occurred during the hybridization process. Moreover, from the acquired results, it can be argued that BZ-9 had faster interfacial charge mobility and separation efficiency. The CV curves of the prepared BZ-9, ZIF-8, ZZ-110, and BiOBr were studied from 0.8 V to −0.8 V, and the results are displayed in Figure 4c. It was obvious that the redox potential of BZ-9 was much higher than BiOBr, ZIF-8, and ZZ-110. The linear sweep voltammetry (LSV) curve revealed that BZ-9 had a low set potential, indicating a lower carrier recombination rate in the catalyst (Figure 4d). In addition, the energy band structures of both BiOBr and ZZ-110 were analyzed via the Mott-Schottky test, as shown in Figure S2. The flat band potential of BiOBr and ZZ-110 relative to the Ag/AgCl reference electrode were −0.117 V and −1.007 V, respectively. According to the following conversion formula:

$$E_{\mathrm{NHE}}{=E}_{\mathrm{Ag}/\mathrm{AgCl}}+E_{\mathrm{Ag}/\mathrm{AgCl}}^{\theta }$$

where $E_{\left(\mathrm{Ag}/\mathrm{AgCl},\;\mathrm{pH}=7\right)}^{\theta }$ = 0.197 V, by converting to a standard hydrogen electrode according to the formula in our previous work [38], the conduction band positions of ZZ-110 and BiOBr were −0.81 and 0.08 eV, respectively.

### 2.2. The Degradation of Wastewater

**Figure 5:**
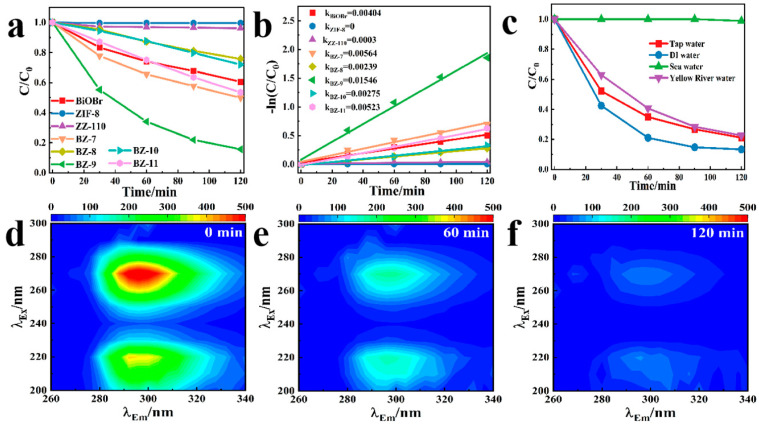
Phenol degradation of the preparation materials (**a**), and the corresponding pseudo first order dynamics curve (**b**); Phenol degradation in natural water bodies by using BZ-9 (**c**); 3D EEMs of Phenol (**d**–**f**).

The performance of photocatalytic materials is an important index for their evaluation. Figure 5 shows the phenol degradation of the prepared catalysts under LED light irradiation. As can be seen from Figure 5a, both ZIF-8 and ZZ-110 had almost no ability to remove phenol, while BiOBr had a certain ability for the photocatalytic degradation of phenol. BZ-9 exhibited the best photocatalytic degradation of phenol, being able to remove nearly 90% of phenol in water within two hours. Figure 5b displays the kinetic constants of the prepared materials, from which it could be observed that the reaction rate of BZ-9 was 3.8 times that of pure BiOBr. Figure 5d–f present a three-dimensional fluorescence spectrum (3D EEMs) that described phenol degradation, in which the PL excitation wavelength of phenol was 270 nm, and its emission wavelength was 296 nm, respectively [39]. It could also be observed that after 120 min of photocatalytic degradation, phenol was almost completely removed.

In order to further study the application of BZ-9 in actual wastewater treatment, several natural water bodies were chosen for phenol removal tests including DI water, tap water, seawater, and Yellow River water, as shown in Figure 5c. It is worth noting that phenol had the fastest degradation rate in DI water, while phenol in seawater exhibited almost no degradation. The degradation of phenol in tap water was also slightly inhibited, possibly due to the interference of anions (i.e., ammonium and chloride ions) [40]. Additionally, the phenol removal rate in the Yellow River water decreased slightly, which might be due to the competitive role of the interfering ions or organic substances in the photocatalytic process [41]. The concentration of phenol in seawater exhibited almost no change, which might be due to the influence of salinity ions in seawater on phenol degradation [42].

**Figure 6:**
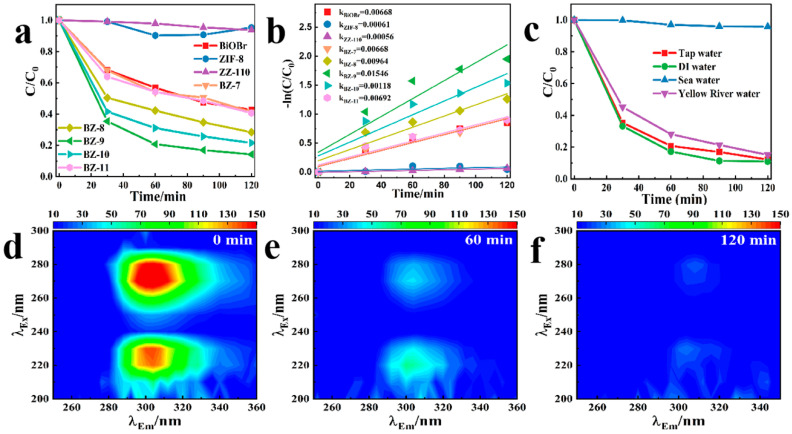
BPA degradation of the preparation materials (**a**), and the corresponding pseudo first order dynamics curve (**b**); BPA degradation in natural water bodies using BZ-9 (**c**); 3D EEMs of BPA (**d**–**f**).

Figure 6 illustrates the prepared catalyst for the degradation of BPA. The removal process of BPA was like that of phenol, and BZ-9 was the best photocatalyst. The kinetic constant of BZ-9 was 2.3 times that of pure BiOBr. Similarly, both ZIF-8 and ZZ-110 had no photocatalytic activity for BPA. Figure 6d–f present 3D EEMs describing the phenol degradation, in which the PL excitation and emission of BPA were about 200 nm and 400 nm, respectively. As could be seen from Figure 6d, the initial BPA signal was very strong. Over time, the fluorescence signal of BPA gradually weakened. After 120 min, only weak fluorescence signals of BPA were detected, indicating that BPA was almost completely removed. The degradation of BPA in real water by BZ-9 was also studied, and the results were like those of phenol. The circulation performance of the catalyst was also an important index to evaluate its performance. The cycle degradation performance of BZ-9 is shown in Figures S4 and S5. After the application of three cycles, the degradation performance of BZ-9 had not decreased significantly, indicating that it had good stability.

### 2.3. Photocatalytic H_2_O_2_ Evolution and Possible Mechanism

**Figure 7:**
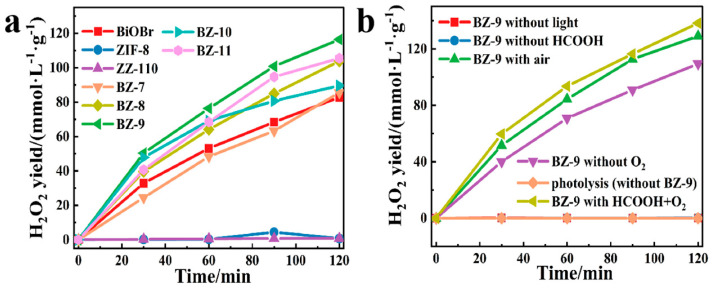
H_2_O_2_ production capacity on prepared materials (**a**); H_2_O_2_ production experiment of BZ-9 under different conditions (**b**).

Figure 7 depicts the H_2_O_2_ yield of the prepared samples under LED lamp illumination. It was found that ZIF-8 and ZZ-110 barely produced H_2_O_2_ (Figure 7a). On the contrary, BiOBr and other composites could produce a certain amount of H_2_O_2_, and the output of H_2_O_2_ increased linearly over a certain period. The production of BZ-9 was the highest within 2 h (116 mmol·L^−1^·g^−1^), and was 1.4 times that of pure BiOBr (82 mmol·L^−1^·g^−1^). Among them, the yields of H_2_O_2_ of BZ-7, BZ-8, BZ-10, and BZ-11 were 85, 103, 89, and 105 mmol·L^−1^·g^−1^ within 2 h, respectively, all of which were less than that of BZ-9. In addition, its recycling capacity should also be studied to evaluate the advantages and disadvantages of the catalyst. The circulation capacity of BZ-9 to generate H_2_O_2_ was shown in Figure S6. After the implementation of four cycles, the H_2_O_2_ production of BZ-9 was only reduced to 109 mmol·L^−1^·g^−1^, indicating that BZ-9 had good stability.

Figure 7b shows the comparison of H_2_O_2_ production under different conditions using a BZ-9 catalyst. As was observed, H_2_O_2_ was minimally produced without light, catalysts, or capture agents. The generation of H_2_O_2_ could be affected to some extent by air exposure and O_2_ entering the reaction system during stirring, however, this was not the main impact. In addition, HCOOH had an important influence on the generation of H_2_O_2_. It was revealed that HCOOH could quickly capture holes and oxidize them to produce HCOO·. The produced HCOO· could further react to form ·OH and H_2_O_2_. Meanwhile, due to the redox potential difference, the electrons of CB at ZZ-110 could reduce O_2_ to H_2_O_2_.

$$h^{+}+\mathrm{HCOOH}\to \mathrm{HCOO}\cdot$$

$${\mathrm{OH}}^{-}+\mathrm{HCOO}\cdot \to \cdot \mathrm{OH}+{\mathrm{HCOO}}^{-}$$

$$2\cdot \mathrm{OH}\to H_{2}O_{2}$$

$$O_{2}+2e^{-}+2H^{+}\to H_{2}O_{2}$$

### 2.4. Mechanism Discussion

To investigate the active groups in the photocatalytic process of BZ-9, quenching, and ESR experiments were conducted. In the quenching experiment, isopropanol (IPA), p-benzoquinone (BQ), and ammonium oxalate (AO) were used as scavengers of ·OH, $\cdot O_{2}^{-}$ and hydroxyl radicals (·OH), respectively. The addition of BQ had the greatest impact on the photocatalytic performance of BZ-9, implying that $\cdot O_{2}^{-}$ radicals play the most important role in the reaction. In addition, IPA also had a certain influence on the photocatalytic reaction when no sacrificial agent existed, indicating that ·OH also has a certain role in promoting the whole degradation process.

**Figure 8:**
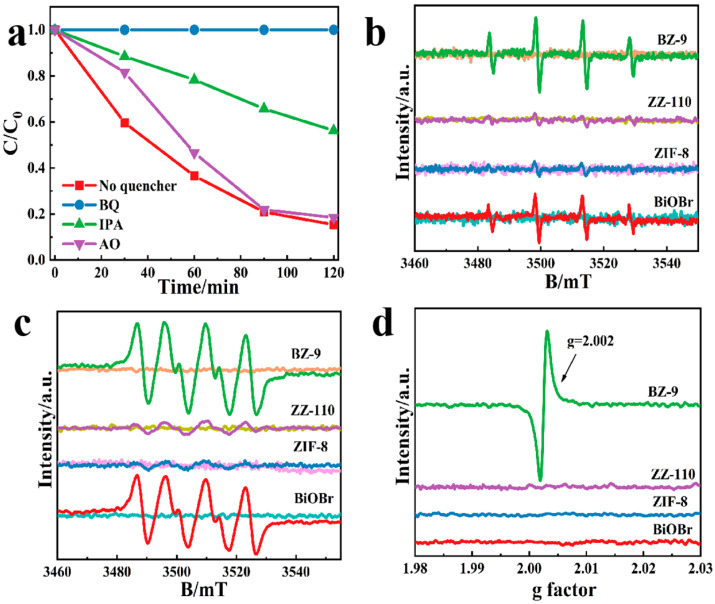
Photocatalytic degradation efficiency of BZ-9 with or without quenching agent under the illumination of LED lamp (**a**); ESR spin-trapping ·OH radicals (**b**); $\cdot O_{2}^{-}$ radicals (**c**), and EPR spectrum of OVs (**d**).

ESR also proved the existence of ·OH radicals and $\cdot O_{2}^{-}$ radicals in BZ-9. Figure 8b depicts the signal diagram of ·OH radicals in the prepared samples. Both BiOBr and BZ-9 had strong signals after illumination, indicating that a mass of ·OH radicals was generated for the degradation of the pollutants. Similarly, $\cdot O_{2}^{-}$ radicals were not observed under dark conditions, but after illumination, $\cdot O_{2}^{-}$ radicals were generated, which corresponded to the quenching experimental conclusion. In addition, from the EPR test, the existence of oxygen vacancy defects was proved. As could be seen from Figure 5d, there were no oxygen vacancies in BiOBr, ZIF-8, or ZZ-110, while BZ-9 generated a strong oxygen vacancy signal at g = 2.002. Thereby, it can be concluded that many oxygen vacancies were introduced during the recombination process, which captured a large number of electrons and produced a strong signal [32].

**Scheme 1:**
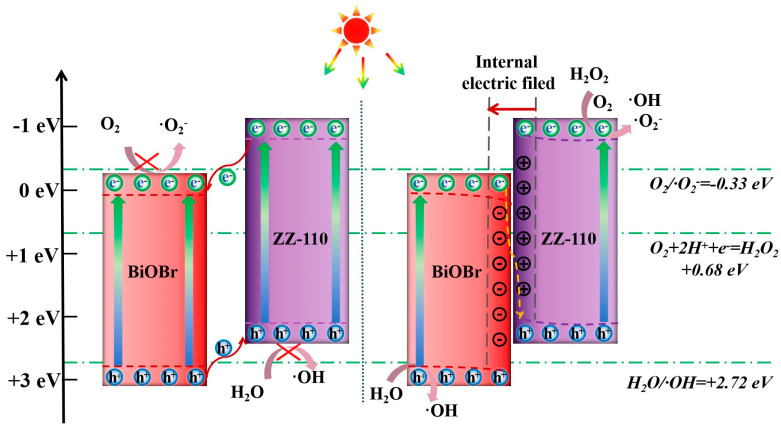
Photocatalytic mechanism of BiOBr and ZZ-110 forming a traditional type II heterojunction and S-scheme heterojunction.

Based on the above-mentioned outcomes, the underlying mechanism of photocatalysis was explored, and two possible mechanisms could be determined. The first photocatalysis mechanism assumed that BiOBr and ZZ-110 form a typical type II heterojunction, as shown in Scheme 1. Under light irradiation, electrons in the valence bands of BiOBr and ZZ-110 were excited to their conduction bands. Moreover, due to the potential difference, the holes located in the valence band of BiOBr migrated to the valence band of ZZ-110, while the electrons on the conduction band of ZZ-110 transferred to the conduction band of BiOBr, which realized the carrier transport separation. However, due to the limitation of redox potential, the holes located in the valence band of ZZ-110 could not react with H_2_O to generate active ·OH radicals, and the electrons located in the conduction band of BiOBr could not reduce O_2_ to $\cdot O_{2}^{-}$ radicals. Thus, the active groups generated by this hole and electron migration pathway were limited and could not degrade wastewater efficiently. Therefore, the traditional type II heterojunction theory was overturned.

$$\mathrm{BiOBr}+\mathrm{hv}\to e_{\mathrm{CB}}^{-}+h_{\mathrm{VB}}^{+}$$

$$\mathrm{ZZ}\text{-}110+\mathrm{hv}\to e_{\mathrm{CB}}^{-}+h_{\mathrm{VB}}^{+}$$

$$h_{\mathrm{VB}}^{+}(\mathrm{BiOBr})\to h_{\mathrm{VB}}^{+}(\mathrm{ZZ}\text{-}110)<E^{\theta }(H_{2}O/\cdot \mathrm{OH})=2.72\;\mathrm{eV}\;\mathrm{vs}.\;\mathrm{NHE}$$

$$e_{\mathrm{CB}}^{-}(\mathrm{ZZ}\text{-}110)\to e_{\mathrm{CB}}^{-}(\mathrm{BiOBr})>E^{\theta }(O_{2}/\cdot O_{2}^{-})=-0.33\;\mathrm{eV}\;\mathrm{vs}.\;\mathrm{NHE}$$

Therefore, BiOBr and ZZ-110 were assumed to be an S-scheme heterojunction. It can be inferred that an internal electric field (IEF) was generated at the interface between BiOBr and ZZ-110. Due to the impact of IEF at the interface, the CB of BiOBr bent downward, the VB of ZZ-110 bent upward, and electrons and holes recombined at the interface. At the same time, the electrons and holes with the higher redox potential on the VB of BiOBr and the CB of ZZ-110 were retained for the subsequent reaction. These holes and electrons with a high redox potential reacted with H_2_O and O_2,_ respectively, to produce ·OH and $\cdot O_{2}^{-}$ radicals and then degraded the pollutants. This theory is supported by XPS, capture experiments, and ESR test results.

$$\mathrm{BiOBr}+\mathrm{hv}\to e_{\mathrm{CB}}^{-}+h_{\mathrm{VB}}^{+}$$

$$\mathrm{ZZ}\text{-}110+\mathrm{hv}\to e_{\mathrm{CB}}^{-}+h_{\mathrm{VB}}^{+}$$

$$e_{\mathrm{CB}}^{-}(\mathrm{BiOBr})\to h_{\mathrm{VB}}^{+}(\mathrm{ZZ}\text{-}110)$$

$$h_{\mathrm{VB}}^{+}(\mathrm{BiOBr})+H_{2}O\to \cdot \mathrm{OH}$$

$$H_{2}O_{2}+\mathrm{hv}\to 2\;\cdot \mathrm{OH}$$

$$e_{\mathrm{CB}}^{-}(\mathrm{ZZ}\text{-}110)+O_{2}\to \cdot O_{2}^{-}$$

$$\cdot O_{2}^{-}/\cdot \mathrm{OH}+\mathrm{pollutant}\to \mathrm{small}\;\mathrm{molecules}\to CO_{2}+H_{2}O$$

## 3. Experimental Section

### 3.1. Preparation of ZIF-8 Catalyst

4.46 mmol of Zn(NO)_3_·6H_2_O (1.19 g) and 8.64 mmol of 2-methylimidazole (1.22 g) were dissolved in 50 mL methanol, respectively. They were then mixed together, and the solution was magnetized for 24 h. The powder obtained by centrifugation was washed three times with methanol and dried at 60 °C. The obtained sample was dried to form ZIF-8.

### 3.2. Preparation of BiOBr/ZIF-8/ZnO Catalyst

**Scheme 2:**
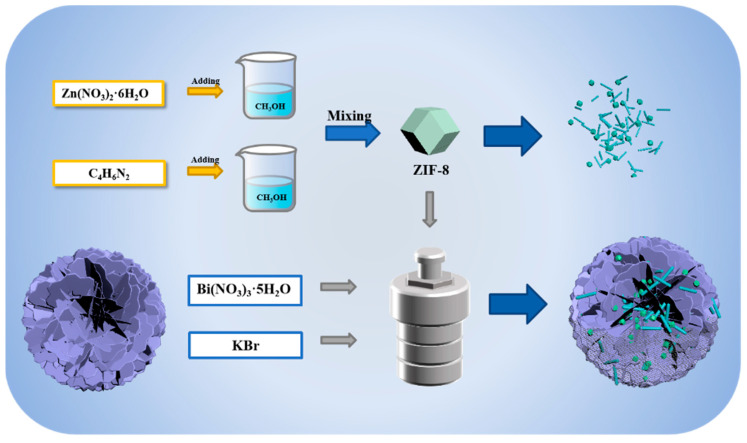
Preparation diagram of BiOBr/ZIF-8/ZnO.

4.8 mmol of Bi(NO)_3_·5H_2_O (2.328 g) and 4.8 mmol of KBr (0.5712 g) were dissolved in a mixed solution of 12 mL H_2_O and 28 mL ethylene glycol and then stirred for several minutes. ZIF-8 was added to the above solution, which was then transferred into a stainless reactor with PTFE lining and held at a temperature of 110 °C for 8 h. The powder obtained by centrifugation was washed three times with DI water and ethanol and dried at 60 °C. The materials with mass ratios of ZIF-8/BiOBr of 7%, 8%, 9%, 10% and 11% were marked as BZ-7, BZ-8, BZ-9, BZ-10 and BZ-11, respectively. Particularly, ZIF-8/ZnO was a compound without Bi(NO)_3_·5H_2_O and KBr, marked as ZZ-110. Preparation diagram of BiOBr/ZIF-8/ZnO shows in Scheme 2, The yield of single catalyst synthesis is shown in Table S1.

### 3.3. Characterization and Photocatalysis Experiment

The instrument parameters and the process of the photocatalytic experiment are shown in the Supplementary Materials.

## 4. Conclusions

In conclusion, ZIF-8 was prepared as the precursor, and ZIF-8/ZnO was formed via the hydrothermal method and embedded in the BiOBr layer to produce BiOBr/ZIF-8/ZnO photocatalyst materials. This combination effectively prevented the agglomeration of ZIF-8, while the separation of electron holes was realized. The prepared BiOBr/ZIF-8/ZnO composite had applications in the degradation of phenol-containing wastewater and the generation of H_2_O_2_. Under LED irradiation, 90% phenol and bisphenol A in wastewater could be effectively removed in 2 h, which was a great improvement compared with BiOBr, ZIF-8, and ZIF-8/ZnO. The mechanism of the electron transport and reaction were systematically examined. BiOBr and ZIF-8/ZnO formed an S-type heterojunction, which helped to effectively separate holes and electrons. By conducting various electron spin resonance measurements, the presence of oxygen vacancy defects in the composite species was corroborated and was found to have a positive impact on photocatalysis in the heterojunction. In addition, it was also found that the BiOBr/ZIF-8/ZnO catalyst had a certain influence on the degradation of phenols in actual wastewater. Our work provides a way for the efficient removal of refractory pollutants and the generation of H_2_O_2_ in the actual water body.

## Data Availability

Not applicable.

## Supplementary Materials

The following supporting information can be downloaded at: https://www.mdpi.com/article/10.3390/molecules28062422/s1, Table S1: Yield of prepared catalyst (average data of three repetitions); Table S2: Comparison of the nitrogen adsorption characteristics of BZ-9, ZIF-8, ZZ-110 and BiOBr; Figure S1: Full spectrum of ZIF-8, ZZ-110 and BiOBr (a); and high-resolution XPS spectra of Zn 4f (b) and O 1s (c).; Figure S2: Mott–Schottky plots of BiOBr (a) and ZZ-110 (b); Figure S3: Nitrogen adsorption-desorption isotherms of the prepared BZ-9, ZIF-8, ZZ-110 and BiOBr (a); pore size distribution curves of BZ-9, ZIF-8, ZZ-110 and BiOBr; Figure S4: Recycling stability of phenol degradation with BZ-9; Figure S5: Recycling stability of BPA degradation with BZ-9; Figure S6: The stability experiments of BZ-9.
