# Supplementary material for: A Bifunctional BiOBr/ZIF-8/ZnO Photocatalyst with Rich Oxygen Vacancy for Enhanced Wastewater Treatment and H2O2 Generation
Source: Molecules. 2023 Mar 7;28(6):2422. doi: 10.3390/molecules28062422 (PMC10056313; doi:10.3390/molecules28062422)
Supplement: Supplementary file 1 [file molecules-28-02422-s001.zip › molecules-2243623-supplementary.pdf]

Supplementary Materials for

A Bifunctional BiOBr/ZIF−8/ZnO Photocatalyst with Rich Oxygen Vacancy for Enhanced Wastewater Treatment and H_2O_2 Generation

Xiao Han, Tianduo Zhang, Yang Cui, Zhaoyang Wang, Ruoyu Dong, Yuhan Wu, Cuiwei Du, Ruyan Chen, Chongfei Yu, Jinglan Feng, Jianhui Sun*, and Shuying Dong*

[1]*School of Environment, Henan Normal University, Key Laboratory for Yellow River and Huai River Water Environmental and Pollution Control, Ministry of Education, Henan Key Laboratory for Environmental Pollution Control, Xinxiang, Henan 453007, China*

* Corresponding author. E-mail: sunjhhj@163.com (J.H. Sun).

* Corresponding author. E-mail: shidashuying@163.com (S.Y. Dong).

2. Experimental

The chemical reagents in this study are further purified. Bismuth nitrate pentahydrate ($Bi(NO_3)_3 \cdot 5H_2O$, ⩾99%), potassium bromide (KBr, ⩾99%), zinc nitrate ($Zn(NO_3)_2 \cdot 6H_2O$, ⩾99%), 2−methylimidazole (⩾98%) and phenol (≥99%) were obtained from Aladdin Reagent Co., Ltd. (Shanghai China). Hydrogen peroxide (H_2O_2, ⩾30%), titanium potassium oxalate ($K_2TiO(C_2O_4)_2$, ⩾98%), ethylene glycol (EG, ⩾99.5%), methyl alcohol (⩾99.5%), and ethanol (⩾99.5%) was purchased from Tianjin Chemical Reagent Co., Ltd, (Tianjin, China).

2.2 Characterization

The crystal morphology of the as−prepared catalysts was analyzed by conducting X−ray diffraction (XRD) measurements using Bruker−D8−AXS diffractometer (Bruker Co., Germany) that was configured with Cu Kα radiation (λ = 0.15406 Å), where the 2θ has a diffraction range of 5−70°. Scanning electron microscopy (SEM, SU8000) was used to record the microscopic morphology of the prepared samples. The chemical structure of the prepared samples was analyzed by carrying out Fourier transform Infrared (FT−IR) spectroscopy (NEXUS) measurements. Ultraviolet−visible diffuse reflectance spectroscopy (UV−vis DR) was detected by using a UV−vis−NIR spectrophotometer (Lambda 950, PerkinElmer) in the spectral range of 300−800 nm, where $BaSO_4$ was employed as a reference. X−ray photoelectron spectroscopy (XPS) (Escalab−250) was utilized to detect the elements and element states of the prepared samples, which were configured with Al Kα (150W). The specific surface area (BET) and the pore structure of the prepared samples were measured with Belsorp−max Surface Area and Pore Size Analyzer (Microtrac BEL) at the temperature value of −196 °C. On top of that, an electron spin resonance spectrometer (Bruker, JES−FA200) was used to measure electron paramagnetic resonance (EPR) and electron spin resonance (ESR) signals in the 5,5−dimethyl−1−pyrroline N−oxide (DMPO) solution at room temperature.

2.3 Electrochemical characterization

The photocatalytic performance of the as−prepared photocatalysts was evaluated by degradation of phenol wastewater and BPA wastewater under the irradiation of a

7W LED lamp. 25 mg of the samples were dispersed in 50 ml of pollutants, and then the mixture solution was adsorbed in the dark for 30 min under magnetic stirring. As a result, so that the catalysts and pollutants reached a state of adsorption−desorption equilibrium. For each 30 min, 4 ml of the supernatant of the mixed solution was analyzed by using a UV−vis spectrophotometer (UV−1700, SHIMADZU). The following calculation formula was used for the degradation efficiency:

$$\text{degradation efficiency (100\%)} = (1 - C_t/C_0) \times 100\% \quad (1)$$

where C_0 denotes the concentration after adsorption−desorption equilibrium, and C_t represents the concentration of pollutants at reaction time t (h).

2.4 Electrochemical activity tests

The optoelectronic properties of the catalysts were tested by using an electrochemical workstation (Zahner Zennium, Germany). More specifically, a traditional three−electrode system was employed, in which the Pt mesh was the counter electrode, the Ag was the reference electrode and Na_2SO_4 solution (0.5 $mol·L^{-1}$) was used as the electrolyte, respectively. The working electrode was obtained by coating the catalyst on the substrate of fluorine−doped tin oxide (FTO). The specific process is as follows: 2 mg of samples and 20 μL of nafion were dispersed in 2 ml of ethanol under ultrasonic conditions, and then the suspension was dropped on 1×1 FTO to dry to be tested. The photocurrent was measured by recording the current change under irradiation with a 300 W xenon lamp. Electrochemical impedance spectroscopy (EIS) was also carried out in the range of 0.01 to 100 kHz by using potentiostatic method (5 mV). Moreover, cyclic voltammetry (CV) and linear sweep voltammetry (LSV) measurements were measured conducted at a scan rate of 50 $mV·s^{-1}$.

2.5 H_2O_2 generation

0.025 g of the catalysts were added into a mixed solution containing 5 mL of HCOOH and 45 mL of H_2O and bubble it to saturation with oxygen (30 min). Subsequently, the mixed solution was allowed to adsorb in the dark for 40 min. After adsorption, the light source (7 W LED) was turned on to take the supernatant every 30 minutes for measurement. The H_2O_2 concentration was then measured at 399 nm by a

UV−vis spectrophotometer (T6, PERSEE) using the potassium titanium oxalate (IV) method.

3. Results and discussion

3.1. Characterization

The BET and pore structure of the prepared BZ−9, ZIF−8, ZZ−110 and BiOBr can be described by nitrogen adsorption−desorption isotherms (Fig. S3). The nitrogen adsorption isotherms of ZIF−8 and ZZ−110 conformed to the type I isotherm (Langmuir isotherm) and belonged to the typical microporous structure. The BET surface area of ZIF−8 was 1174.7 $m^2 \cdot g^{-1}$, while the BET surface area of ZZ−110 was greatly reduced due to the presence of ZnO. What's more, the nitrogen adsorption isotherms of BiOBr conformed to the type Ⅱ isotherm (Brunauer−Deming−Deming−Teller−classification). Since the content of ZZ−110 in BZ−9 was less, the adsorption curve and pore size distribution of BZ−9 were similar to that of BiOBr. Compared with pure BiOBr, the BET surface area and pore diameter of BZ−9 increased to a certain extent. Moreover, the BET surface area, pore diameter and pore volume data are shown in Table S2, ZIF−8 and ZZ−110 were microporous materials, and BiOBr and BZ−9 were mesoporous materials.

Table S1 Yield of prepared catalyst (average data of three repetitions).

Sample	Amount of ZIF	yields of catalyst (%)
BOB	/	85.43
ZIF	/	82.36
ZZ−110	/	89.83
BZ−7	0.078 g	85.93
BZ−8	0.089 g	85.15
BZ−9	0.1 g	84.45
BZ−10	0.11 g	84.15
BZ−11	0.12 g	86.79

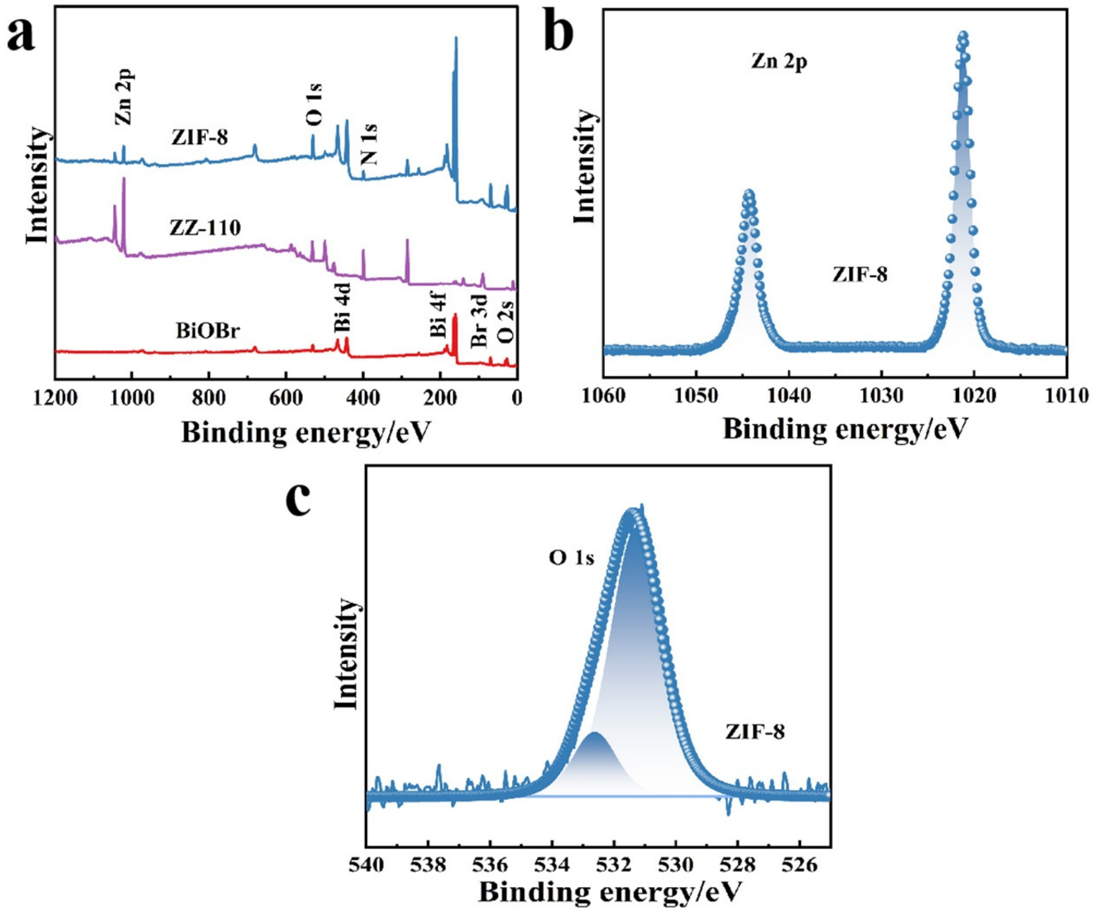

Fig. S1. Full spectrum of ZIF−8, ZZ−110 and BiOBr (a); and high−resolution XPS spectra of Zn 4f (b) and O 1s (c).

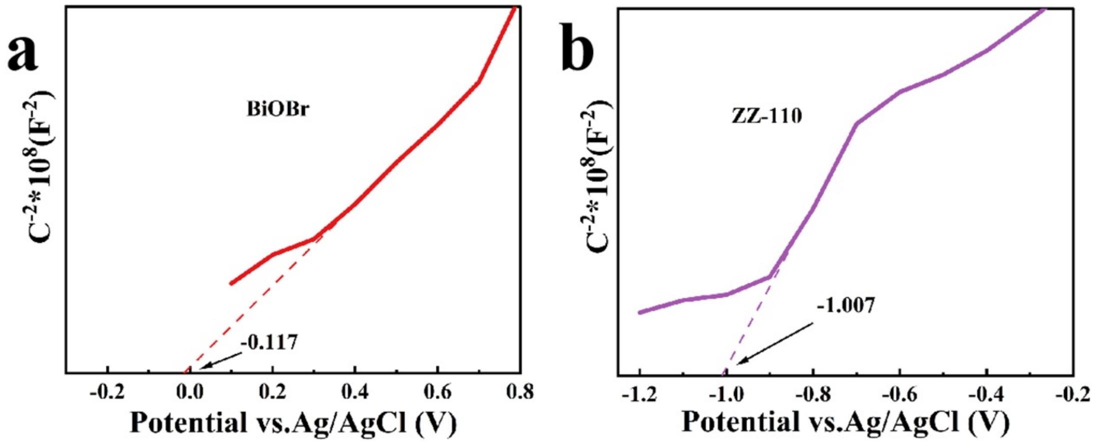

Fig. S2. Mott–Schottky plots of BiOBr (a) and ZZ−110 (b).

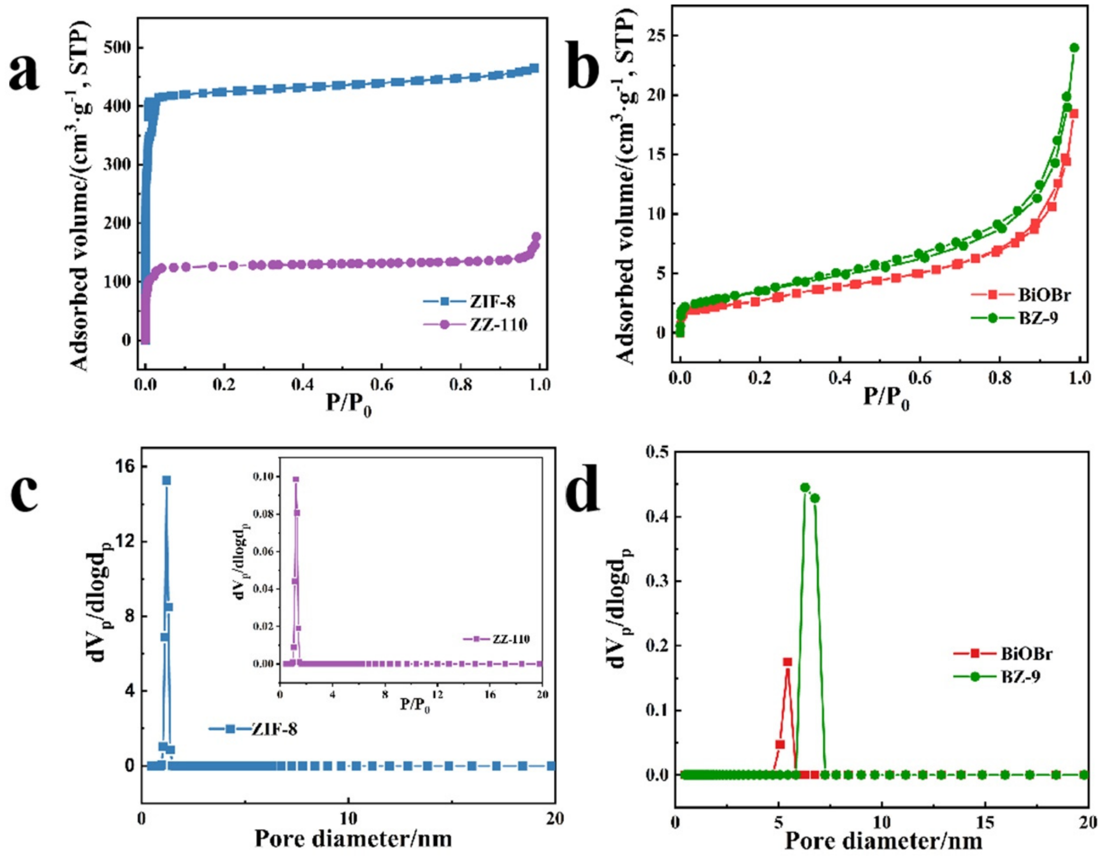

Fig.S3. Nitrogen adsorption−desorption isotherms of the prepared BZ−9, ZIF−8, ZZ−110 and BiOBr (a); pore size distribution curves of BZ−9, ZIF−8, ZZ−110 and BiOBr.

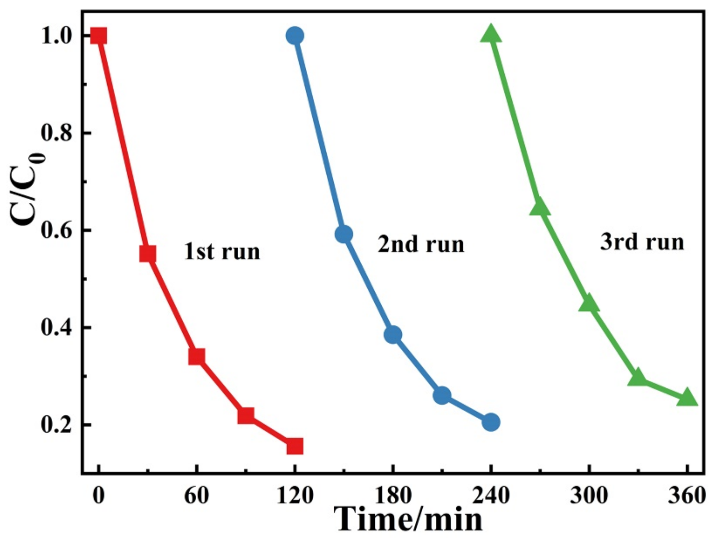

Fig.S4. Recycling stability of phenol degradation with BZ−9

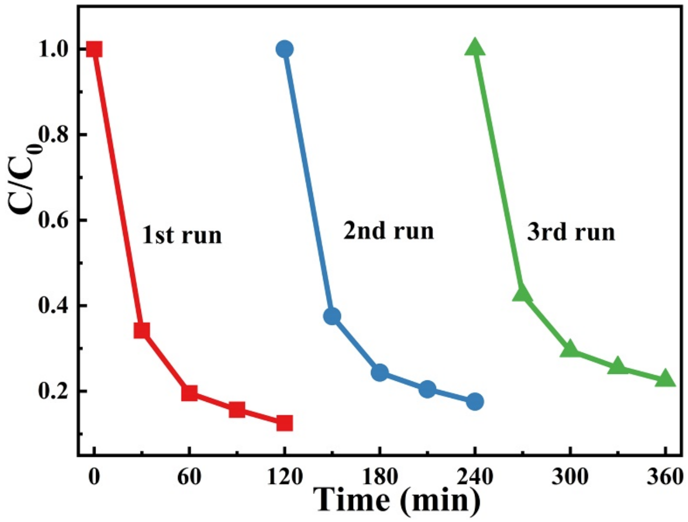

Fig.S5. Recycling stability of BPA degradation with BZ−9

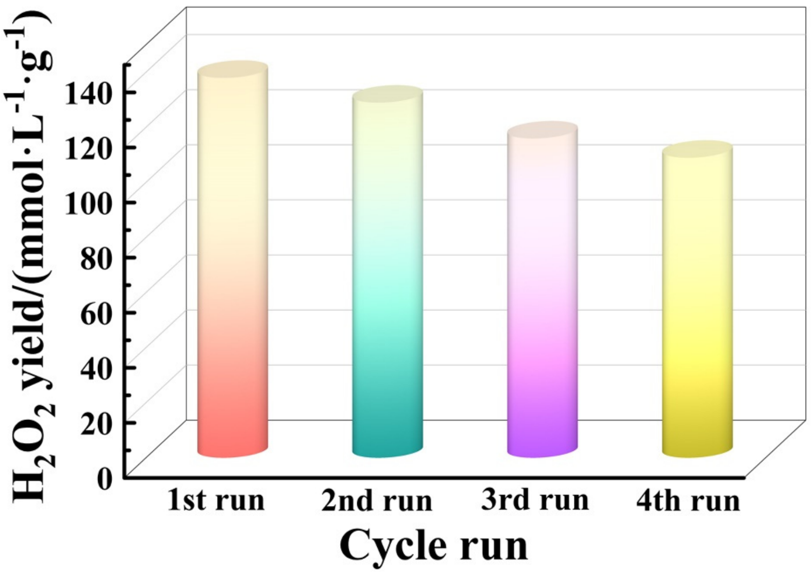

Fig.S6. The stability experiments of BZ−9

Table S2. Comparison of the nitrogen adsorption characteristics of BZ−9, ZIF−8, ZZ−110 and BiOBr.

Sample	BET surface area[a] / $m^2 \cdot g^{-1}$	Pore diameter[c] / nm	Pore volume[b] / $cm^3 \cdot g^{-1}$
ZIF−8	1174.7	2.4484	2.4484
ZZ−110	442.04	2.3625	2.3625
BiOBr	11.59	9.8368	9.8368
BZ−9	13.557	10.938	10.938

[a] BET surface area calculated from the linear portion of the BET plot (P/P_0 = 0.29)

[b] Total pore volume, taken from the volume of N_2 adsorbed at P/P_0=0.99

[c] Average pore diameter estimated using the desorption branch of the isotherm and the Barrett−Joyner−Halenda (BJH) formula.
